# The use of immersive virtual reality for cancer-related cognitive impairment assessment and rehabilitation: A clinical feasibility study

**DOI:** 10.1016/j.apjon.2022.100079

**Published:** 2022-05-17

**Authors:** Yingchun Zeng, Linghui Zeng, Andy S.K. Cheng, Xijun Wei, Boran Wang, Jingchi Jiang, Jin Zhou

**Affiliations:** a School of Medicine, Zhejiang University City College, Hangzhou, China; b Department of Rehabilitation Sciences, The Hong Kong Polytechnic University, Hong Kong SAR, China; c Department of Rehabilitation Medicine, Shenzhen Hospital, Southern Medical University, Shenzhen, China; d Department of Computer Sciences, Harbin Institute of Technology, Shenzhen, China; e Department of Computer Sciences, Harbin Institute of Technology, Harbin, China; f Department of Nursing, Integrated Hospital of Traditional Chinse Medicine, Southern Medical University, Guangzhou, China

**Keywords:** Immersive VR, Cognitive assessment, Rehabilitation intervention, Chinese cancer patients

## Abstract

**Objective:**

This brief study aimed to examine the potential effects of virtual reality (VR)-assisted cognitive rehabilitation intervention on the health outcomes of patients with cancer.

**Methods:**

A single group of pre-test and post-test study designs were used. An innovative VR system was developed to assess cancer-related cognitive impairment and provide cognitive rehabilitation. The potential effects of the system were determined by measuring changes in cognitive function (learning and memory, information processing speed, executive function, and verbal fluency) and the severity of depression, anxiety, and insomnia.

**Results:**

Nine subjects completed the entire VR intervention and were included in the analysis. The participants’ mean age was 43.3 years (standard deviation, 8.9 years). The VR-based cognitive intervention significantly improved the subjective cognitive measures of perceived cognitive impairment and perceived cognitive ability (*P* ​= ​0.01 and *P* ​< ​0.01, respectively). The intervention also improved the objective cognitive measures of verbal learning memory as measured using the Auditory Verbal Learning Test (eg., *P* ​< ​0.01 for 5-min delay recall), information processing speed as measured using the trail-making test-A (*P* ​= ​0.02) and executive function as measured using the trail-making test-B (*P* ​= ​0.03). Only the subtest of delayed recall showed no statistically significant difference after the intervention (*P* ​= ​0.69). The VR-based psychological intervention significantly reduced the severity of sleep disorders (*P* ​< ​0.01).

**Conclusions:**

The use of immersive VR was shown to have potential effects on improving cognitive function for patients with cancer. Future studies will require a larger sample size to examine the effects of immersive VR-assisted cognitive rehabilitation on the health outcomes of patients with cancer.

## Introduction

In recent decades, cancer-related cognitive impairment (CRCI) has been frequently reported in patients with brain tumors and non-central nervous system cancers.^1^, ^2^, ^3^ The prevalence of CRCI ranges from 21% to 75%.^1^ Moreover, CRCI negatively affects cancer survivors’ working ability, social and occupational functioning, and daily life, and eventually decreases their quality of life.^1^^,^^3^, ^4^, ^5^ Commonly impaired domains of cognition in patients with cancer include attention/concentration, verbal memory, and executive function, resulting in difficulties in engaging in multitasking, low information processing speed, and language problems.^1^^,^^6^^,^^7^

With recent and ongoing advances in technology, the early detection of cognitive decline and the provision of effective interventions are likely to be feasible with the assistance of artificial intelligence (AI).^8^ Of the available AI technologies, virtual reality (VR) is an optimal option for use in cognitive assessment and rehabilitation as it enables the assessment of human behaviors under more ‘natural’ conditions. VR ensures an accurate recording of real-time data and the number of errors made, without having to rely on a researcher's subjective assessments and note-taking. In addition, it provides insight into individuals' typical behaviors. Participants can engage in a ‘real-life’ environment and thus increase their level of participation.^9^ Cognitive assessments using VR currently exist,^10^, ^11^, ^12^ but VR-based neuropsychological assessments are not immersive virtual environments and therefore provide only limited levels of distraction.^12^^,^^13^

In addition, immersive VR has been identified as a potentially revolutionary tool for neuropsychological interventions.^14^ Cognitive interventions assisted by VR technology may be able to reduce CRCI and improve quality of life of patients with cancer. Although Zeng et al^15^ found that VR is widely used in the management of cancer symptoms, such as pain, anxiety, depression, and fatigue, very few studies have used VR to treat CRCI. An ongoing trial is incorporating cutting-edge VR technologies into cognitive training using VR-assisted attention-restorative therapy to treat CRCI in cancer survivors,^16^ but that study does not include a comprehensive VR-assisted assessment of cognition.

Therefore, this study examined the potential effects of a VR-assisted cognitive rehabilitation intervention on the health outcomes of patients with cancer and with subjective cognitive impairment.

## Methods

### Study design

This study used a one-group pre-test and post-test design to examine the feasibility of an innovative VR system for assessing CRCI and providing cognitive rehabilitation. Ethical approval was obtained from the Human Ethics Committee of the Cancer Center of Southern Medical University (Approval No. 202111-K2). All research participants voluntarily joined the study and were able to withdraw at any time. Written informed consent was obtained from each research participant.

### Participants

Study participants were adults aged 18–65 years who had had a primary cancer diagnosis. All participants had been admitted to the Cancer Center of Southern Medical University for treatment. Because the VR-assisted cognitive assessment system was recently developed, the optimal cut-off value for detecting CRCI was unknown. Thus, the perceived cognitive impairment (PCI) and perceived cognitive ability (PCA) subscales of the functional assessment of cancer therapy—cognitive function (FACT-Cog), version 3 were used to identify patients with cancer and with cognitive impairment.^17^ The FACT-Cog has been widely used to assess Chinese patients with cancer, and its psychometric properties have shown excellent reliability and validity.^18^ The PCI subscale includes 18 items, each of which needs reverse scoring, whereas the PCA subscale is composed of seven items, none of which requires reverse scoring. Higher scores in both subscales indicate better cognitive function. Subjective CRCI was defined by using cut-off scores of PCI ≤ 44 or PCA ≤ 9.^19^

### Assessment and intervention

The study's design and development processes were iterative and were performed in close collaboration with a VR technology company. In the assessment development phase, the recommendations of the International Cognition and Cancer Task Force (ICCTF) were followed. Key domains of cognitive impairment in patients with cancer are learning and memory, information processing speed, executive function, and verbal fluency.^6^ Hence, the VR-assisted cognitive assessment system created the following three virtual environments to assess the key cognitive domains of CRCI: (1) an auditory verbal learning test (AVLT) to assess verbal learning and memory, (2) a verbal fluency test (VFT) to assess verbal fluency, and (3) a trail-making test-A (TMT-A) to assess information processing speed and a trail-making test-B (TMT-B) to assess executive functioning. The details of the neurocognitive tests AVLT, TMT, and VFT have been described previously.^20^^,^^21^

**Fig. 1:**
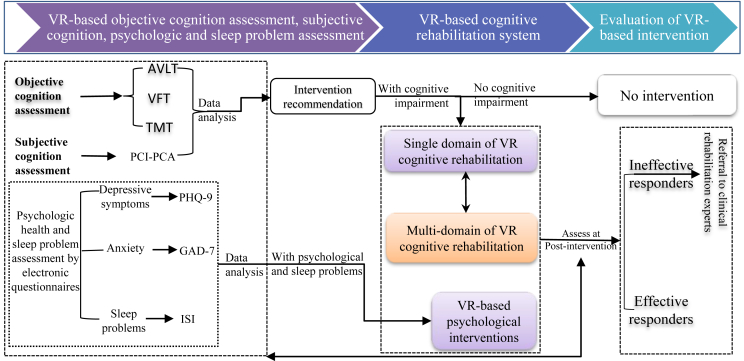
Design framework of CRCI assessment and rehabilitation system (Part of this system has been grated as National Invention Patent: #202110746318.4). AVLT, auditory verbal learning test; CRCI, cancer-related cognitive impairment; GAD-7, seven-item general anxiety disorder; ISI, Insomnia Severity Index; PHQ-9, nine-item Patient Health Questionnaire; TMT, trial making test; VFT, verbal fluency test.

**Fig. 2:**
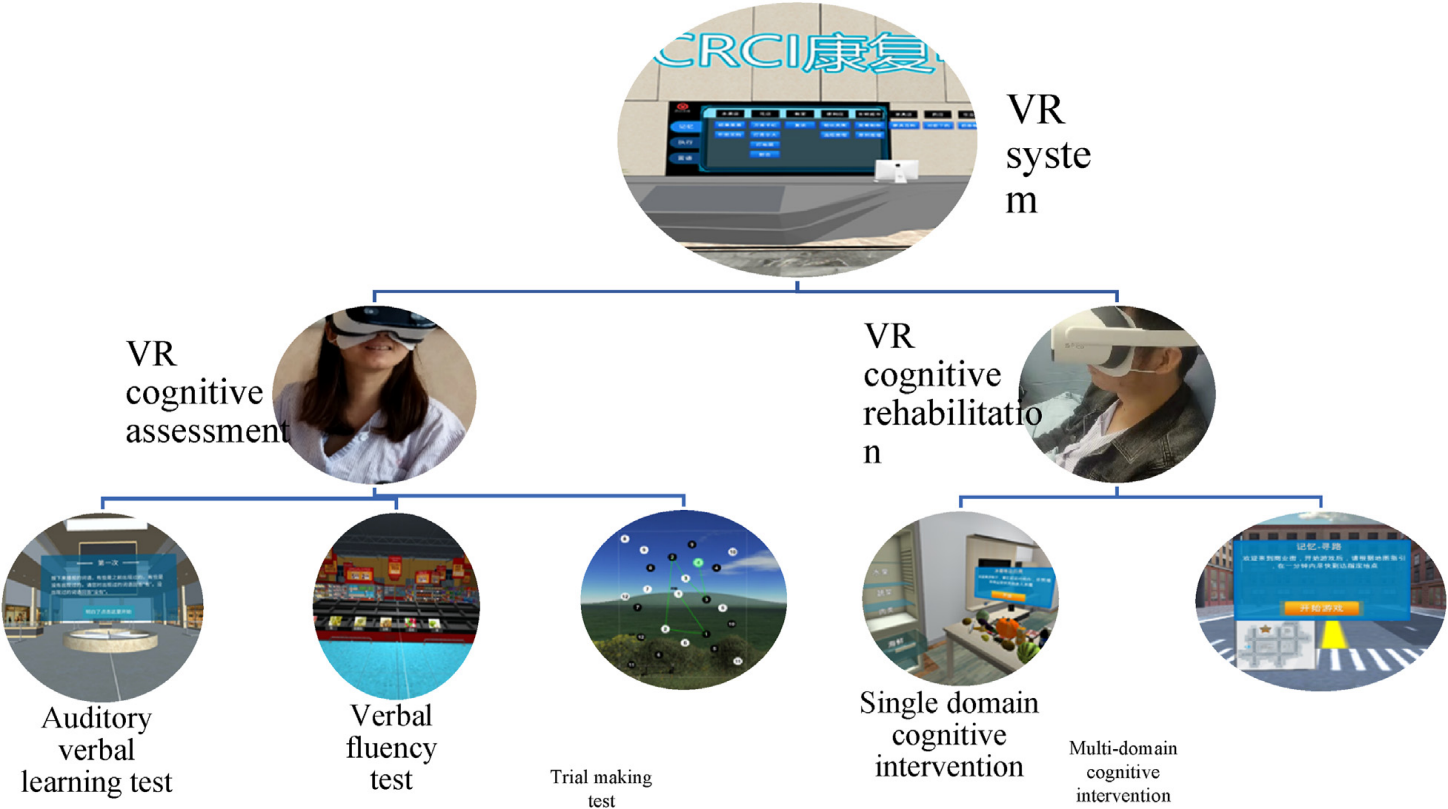
Immersive virtual reality system scenarios.

Because patients with cancer may have CRCI in either a single domain or multiple domains of cognition, the VR cognitive rehabilitation system was designed with a single domain and multidomain cognitive rehabilitation capabilities. Fig. 1 shows the entire study design framework and Fig. 2 shows the scenarios used for the VR assessment and the VR cognitive intervention. The intervention was designed to be administered in approximately ten 30-min VR sessions over a period of 2 weeks. However, due to the different durations of hospitalization amongst the participants, the study allowed for a degree of flexibility in intervention length, and some patients were offered two sessions of the intervention per day over a period of 1 week. The post-intervention assessment was performed after the final intervention session.

### Other outcome measures

Because patients with cancer commonly experience anxiety, depression and sleep disorders, depressive symptoms were assessed using the nine-item Patient Health Questionnaire (PHQ-9).^22^ The total possible PHQ-9 scores range from 0 to 27, with higher scores indicating higher levels of depressive symptoms. Anxiety symptoms were assessed using the seven-item general anxiety disorder scale (GAD-7),^23^ with total possible GAD-7 scores ranging from 0 to 21 and higher scores indicating higher levels of anxiety. Chinese versions of PHQ-9 and GAD-7 had acceptable reliability and validity among patients with cancer.^24^ The Insomnia Severity Index (ISI) was used to assess sleep disorders of patients with cancer, with a cut-off score of 10 used to detect cases of insomnia^25^ (a total ISI score greater than 10 indicates that the patient has sleep disorders). The Chinese version of ISI was validated among patients with cancer and reported good psychometric properties.^26^

### Data ccollection pprocedure

All research participants voluntarily joined this study and gave written informed consent. Data were collected by a research nurse from November 2021 to February 2022. All the outcome measures were assessed at baseline and post-intervention.

### Data analysis

Data analyses were performed using SPSS statistical software version 25 (IBM, Armonk, NY, USA). Descriptive statistics and paired Student's *t*-tests were used to describe the characteristics of the study participants and analyze the potential effects of the VR intervention at baseline and post-intervention.

## Results

A total of 89 subjects were screened. Of these subjects, 17 were determined to have a cognitive impairment, and nine subjects completed the entire VR intervention. Eight subjects did not complete all of the intervention sessions due to the following reasons: a heavy treatment burden (*n* ​= ​1), significant disease progression and deterioration (*n* ​= ​2), severe treatment side-effects due to chemotherapy (*n* ​= ​2), and a need to discharge from hospital because of medical insurance coverage requirements (*n* ​= ​3). There were no statistically significant differences in demographic or clinical characteristics between those who complete the entire VR rehabilitation intervention and those who did not. Only the nine participants who completed the entire intervention were included in the analysis.

The demographic and clinical characteristics of the study sample were collected from a medical record system and are summarized in Table 1. Table 2 shows the differences between the baseline and post-intervention cognitive assessment, psychological health, and insomnia outcomes for the participants.

**Table 1:** Demographic characteristics of the participants (*N* ​= ​9).

Variables	*n* (%)	Mean	SD
**Age (years)**		43.3	8.9
**Gender**			
Male	4 (44.4)		
Female	5 (55.6)		
**Education**			
Primary school	7 (77.8)		
High school	1 (11.1)		
University or above	1 (11.1)		
**Employment status**			
Employed	3 (33.3)		
Unemployed	6 (66.7)		
**Marital status**			
Married	8 (88.9)		
Unmarried	1 (11.1)		
**Disease diagnosis**			
Brain tumor	3 (33.3)		
Liver cancer	3 (33.3)		
Breast cancer	2 (22.2)		
Lung cancer	1 (11.1)		
**Disease stage**			
Early stage	6 (66.7)		
Advanced stage	3 (33.3)		
**Treatment types**			
Surgery only	2 (22.2)		
Surgery ​+ ​Chemotherapy	4 (44.4)		
Surgery ​+ ​Chemotherapy ​+ ​Radiotherapy	3 (33.3)

**Table 2:** Differences of outcome measures at baseline and post-intervention (*N* ​= ​9).

Variables (Total score ranges)	Baseline, Mean (SD)	Post-intervention, Mean (SD)	*t*	*p*
**PCI** (0–72)	43.33 (3.16)	49.00 (4.61)	−3.20	0.01
**PCA** (0–28)	9.33 (1.50)	12.44 (3.67)	−3.63	< 0.01
**GAD**-7 (0–21)	4.00 (2.87)	2.77 (2.05)	1.61	0.15
**ISI** (0–28)	11.11 (4.16)	6.88 (4.13)	4.99	< 0.01
**PHQ-9** (0–27)	6.22 (3.99)	3.78 (1.39)	1.72	0.13
**VR-cognition assessment**				
**AVLT**-1st time immediate recall (0–12)	2.22 (1.20)	5.55 (3.16)	−4.00	< 0.01
**AVLT**-2nd time immediate recall (0–12)	4.33 (2.44)	6.88 (3.14)	−2.55	0.03
**AVLT**-3rd time immediate recall (0–12)	5.44 (3.00)	9.00 (2.64)	−6.71	< 0.01
**AVLT**-5-min delay recall (0–12)	5.33 (3.71)	8.44 (2.50)	−4.13	< 0.01
**AVLT**-20-min delay recall (0–12)	6.77 (2.33)	7.33 (3.53)	−0.40	0.69
**AVLT**-recall by classification (0–12)	4.66 (3.12)	9.44 (1.94)	−3.21	0.01
**AVLT**-recall by reorganization (0–24)	21.22 (1.30)	22.22 (1.09)	−1.73	0.12
**VFT**1 (more named animals within a min being better)	9.33 (5.05)	9.55 (5.93)	−0.19	0.85
**VFT**2 (more named vegetables within a min being better)	6.89 (4.16)	7.33 (3.39)	−0.56	0.59
**VFT**3 (more named fruits within a min being better)	7.11 (2.80)	7.00 (2.69)	0.12	0.91
**TMT**-Part A (lower time being higher processing speed)	99.48 (59.94)	76.23 (47.88)	2.97	0.02
**TMT**-Part B (lower time being better executive function)	128.13 (81.28)	79.90 (32.46)	2.76	0.03

AVLT, Auditory Verbal Learning Test; GAD-7, seven-item General Anxiety Disorder; ISI, Insomnia Severity Index; PCA, Perceived Cognitive Ability; PCI, Perceived Cognitive Impairment; PHQ-9, nine-item Patient Health Questionnaire; TMT, Trial Making Test; VFT, Verbal Fluency Test.

As shown in Table 2, the VR-based cognitive intervention significantly improved the patients' subjective cognition, as indicated by their FACT-Cog PCI and PCA subscale scores (*P* ​= ​0.01 and *P* ​< ​0.01, respectively). The VR-based cognitive intervention also improved the patients’ objective cognition, as indicated by improved verbal learning memory as measured using the AVLT, information processing speed as measured using the TMT-A (*P* ​= ​0.02), and executive function as measured using the TMT-B (*P* ​= ​0.03). Only the subtest of delayed recall showed no significant difference after the VR intervention. Finally, the VR psychological intervention significantly reduced the severity of sleep disorders (*P* ​< ​0.01).

## Discussion

### General findings

In this study, a novel CRCI assessment and rehabilitation system guided by the ICCTF guidelines were developed. Using VR technology, research participants were immersed in cognitive intervention tasks that were similar to those experienced in their daily life activities but presented within a VR environment. This format is expected to increase the ecological validity of cognitive assessments and rehabilitation interventions. Traditional paper-and-pencil approaches to cognitive tests lack ecological validity (ie., they do not take environmental factors into account), and thus, they may not reflect the actual cognitive functioning of the participants.^27^ Indeed, previous preliminary results have shown that cancer patients prefer to undergo cognition assessments through a VR-based approach, rather than a paper-and-pencil approach.^28^ Notably, this feasibility study also found potential effects of using immersive VR neuropsychological rehabilitation for improving cognitive function, psychological health, and insomnia in patients with cancer.

The benefits observed in this study were likely due to the immersion effect because VR technology provides a digital simulation of a real environment during cognitive training. Cognitive training delivered by VR stimulates an individual's nervous system and thereby capitalizes on neuroplasticity to promote cognitive rehabilitation.^29^ A VR-based cognitive assessment and rehabilitation system has previously been shown to be difficult to implement in aging patients with cancer,^30^ therefore, the VR rehabilitation system in this study had undergone refinements to address this issue. With the advances in AI technology in recent years, more-immersive VR, better-augmented VR, and mixed VR modalities now enable patients to become more immersed in the virtual environment,^31^ resulting in increased patient compliance and greater beneficial effects of the interventions.

### Limitations

Because this was a clinical feasibility study, it had several limitations. The study was conducted over a short period of time, and thus, we were only able to recruit a limited number of eligible subjects for the VR-based cognitive intervention. In the next stage of the main study, larger sample size will be enrolled to confirm the positive effects of VR interventions on CRCI and psychological health. Moreover, the dropout rate in this study was 50% which may have caused attrition bias. In addition, the nine participants in this study varied in their cancer diagnoses which included brain tumors and non-central nervous system cancers as well as their treatment stages. A future standardized clinical trial will need to enroll subjects that are homogeneous in terms of their cancer type and cancer treatment. Finally, this feasibility study lacked a control group and included only a single group assessed at baseline and post-intervention. Therefore, the findings of this study need to be interpreted in light of these biases.

Despite these limitations, this feasibility study indicated that VR-assisted cognitive assessment and rehabilitation, especially when assisted by immersive VR technology, may have potential benefits for the management of CRCI. As CRCI is a common sequela resulting from cancer itself and from cancer treatment, the use of AI technologies such as immersive VR is warranted to reduce the occurrence of cancer-related symptoms and improve the quality of cancer care and patient satisfaction.

### Implications for nurses

Nurses are uniquely positioned to influence how technology is used at the point of care.^32^ Oncology nurses play key roles in every facet of cancer patient care, from the cost of care to the overall patient experience. AI technologies, such as VR, are emerging as valuable tools for nursing professionals to help implement assessments and interventions, assist with clinical decisions, and ultimately, improve the quality of patient care.^32^ Therefore, educating oncology nurses on how to best interact with VR technologies will provide them with a wide range of solutions for improving the cost-effectiveness of care, the quality of care, and the overall transformation of cancer healthcare.^32^

## Conclusions

This study examined the feasibility of a novel assessment and rehabilitation system for CRCI, as guided by the ICCTF guidelines. Immersive VR also has potential benefits in terms of improving neuropsychological outcomes for patients with cancer. Future studies will require a larger sample size to fully examine the effects of immersive-VR-assisted cognitive rehabilitation on the health outcomes of patients with cancer.

## Authors contributions

Conceived and designed the analysis: YZ, LZ, ASKC, JZ. Collected the data: YZ, XW, JZ. Contributed data or analysis tools: YZ, BW, JJ. Performed the analysis: YZ, ASKC, BW, JJ. Wrote the paper: YZ, LZ.

## Declaration of competing interest

None declared.

## Funding

This study was funded by the 10.13039/501100001809National Natural Science Foundation of China (Grant No. 72004039) for Dr Zeng, and by the Research Area Based Seed Fund by the Department of Rehabilitation Sciences, The 10.13039/501100004377Hong Kong Polytechnic University (ZVRQ) for Dr Cheng.

## Ethics statement

This study was approved by the Human Ethics Committee of the Cancer Center of Southern Medical University (Approval No. 202111-K2). Written informed consent was obtained from each research participant.
