# Supplementary material for: The use of immersive virtual reality for cancer-related cognitive impairment assessment and rehabilitation: A clinical feasibility study
Source: Asia Pac J Oncol Nurs. 2022 May 17;9(12):100079. doi: 10.1016/j.apjon.2022.100079 (PMC9579330; doi:10.1016/j.apjon.2022.100079)
Supplement: Multimedia component 1 [file mmc1.docx]

**Appendix 1. Technical parameters for developing VR cognition assessment and rehabilitation system**

| Hardware development environment | i7-8700 processor, GTX 1060, 32 GB Memory, 512 GB Hard Disk Drive |
| --- | --- |
| Hardware-operating environment | 1. PC：i7-8700 processor, 32 GB Memory, 512 GB Hard Disk Drive 2. PICO Neo 2 VR headsets |
| Software development environment and running system | Windows10、Linux |
| Software development environment or development tools | Unity3D、Visual Studio、and PHP |
| The software running platform or the operating system | Windows and Linux system  PICO Neo 2 VR headsets |
| Software running support environment or supporting software | IE Browser,360 Browser, Firefox Browser, Google Browser, PHP7.3、Ngix1.18、.NET Framework4.0、MySQL5.7 |
| Programming language | C#、PHP、HTML、JS、CSS |
